# Supplementary material for: 2-(4-Benzyloxy-3-methoxyphenyl)-5-(carbethoxyethylene)-7-methoxy-benzofuran, a Benzofuran Derivative, Suppresses Metastasis Effects in P53-Mutant Hepatocellular Carcinoma Cells
Source: Biomedicines. 2023 Jul 19;11(7):2027. doi: 10.3390/biomedicines11072027 (PMC10377018; doi:10.3390/biomedicines11072027)
Supplement: Supplementary file 1 [file biomedicines-11-02027-s001.zip › biomedicines-2444775-Supplementary.pdf]

HepG2

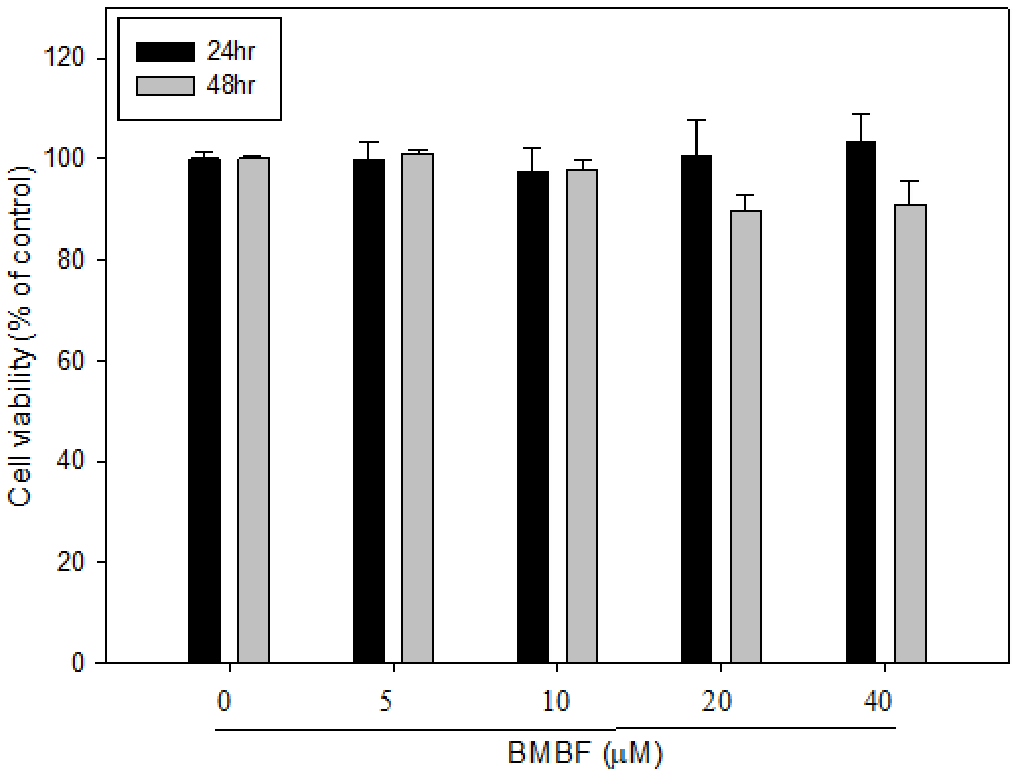

figure S1. BMBF cannot affect the cell viability in HepG2. The cytotoxic effect of BMBF on the HepG2 hepatoblastoma cells was measured by MTT assay. Cells were seeded onto 24-well plates at 2x10^4 cells/well. After attachment, cells were treated with various concentrations of BMBF for 24 and 48 h. The cell viability were determined by MTT assay. The optical density was measured at 563 nm. Data were represented as means ±SD from three independent experiments. The results showed that BMBF cannot affect the cell viability in HepG2.

AML12 mouse hepatocyte

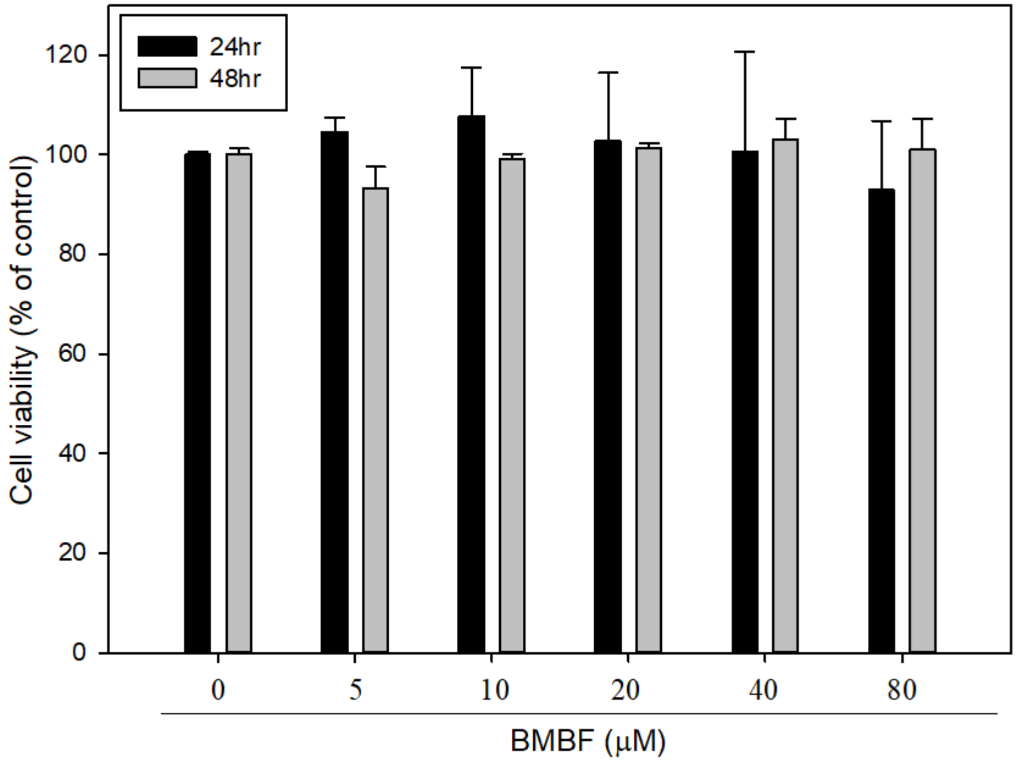

figure S2. BMBF cannot affect the cell viability in normal hepatocytes. AML12 mouse hepatocytes were obtained from Bioresources Collection and Research Center (BCRC, Hsinchu, Taiwan). Cells were grown in DMEM/F12 medium supplemented with 10% FBS, 1X ITS (10 μg/mL insulin, 5.5 μg/mL transferrin, 5 ng/mL selenium), 14 mM sodium bicarbonate, 2.5 mM glutamine, 0.2 mM sodium pyruvate, 15 mM HEPES, 40 ng/mL dexamethasone, 100 units/mL penicillin, and 100 μg/mL streptomycin, pH 7.2. A total of 10^5 cells was seeded onto 24-well culture plates. After treatment with various concentrations of BMBF for 24 and 48 h, the viable cells were determined by MTT assay. The optical density was measured at 563 nm. Data represented as means ±SD (n=3). The results showed that BMBF cannot affect the cell viability in normal hepatocytes.
